# Plant resistance does not compromise parasitoid-based biocontrol of a strawberry pest

**DOI:** 10.1038/s41598-020-62698-1

**Published:** 2020-04-03

**Authors:** Daniela Weber, Paul A. Egan, Anne Muola, Lars E. Ericson, Johan A. Stenberg

**Affiliations:** 1 Department of Plant Protection Biology, Swedish University of Agricultural Sciences, Box 102, 23053 Alnarp, Sweden; 2 Biodiversity Unit, University of Turku, 20014 Turku, Finland; 3 Department of Ecology and Environmental Science, Umeå University, 90187 Umeå, Sweden

**Keywords:** Agroecology, Ecosystem services, Entomology

## Abstract

Plant nutritional  quality can influence interactions between herbivores and their parasitoids. While most previous work has focused on a limited set of secondary plant metabolites, the tri-trophic effects of overall phenotypic resistance have been understudied. Furthermore, the joint effects of secondary and primary metabolites on parasitoids are almost unexplored. In this study, we compared the performance and survival of the parasitoid species *Asecodes parviclava* Thompson on wild woodland strawberry (*Fragaria vesca* L.) genotypes showing variation in resistance against the parasitoid’s host, the strawberry leaf beetle (*Galerucella tenella* L.). Additionally, we related the metabolic profiles of these plant genotypes to the tritrophic outcomes in order to identify primary and secondary metabolites involved in regulating plant potential to facilitate parasitism. We found that parasitoid performance was strongly affected by plant genotype, but those differences in plant resistance to the herbivore were not reflected in parasitoid survival. These findings could be explained in particular by a significant link between parasitoid survival and foliar carbohydrate levels, which appeared to be the most important compounds for parasitism success. The fact that plant quality strongly affects parasitism should be further explored and utilized in plant breeding programs for a synergistic application in sustainable pest management.

## Introduction

In order to protect themselves from insect attack, plants have developed multiple defensive mechanisms which can be grouped into two principal defensive strategies: (1) direct defence, by nutritional unsuitability, deterrence, or toxicity to the herbivore^1,2^; and (2) indirect defence by exploitation of the herbivore’s natural enemies (predators and parasitoids) as bodyguards^3,4^. However, by deploying both strategies simultaneously there is potentially an ecological conflict for the plant where these strategies interact or trade-off^5–7^. In particular, the wide variety of chemical compounds plants produce to combat insect herbivores may also impair the performance of the predators and parasitoids that attack the insect herbivores^8–10^. Research on direct and indirect defences has previously taken mainly separate paths and, thus, knowledge gaps remain on the relationships between these two principal defence strategies.

In the context of sustainable pest control and agroecosystem management, the potential conflict between direct and indirect  defences is of increasing importance. This potential conflict determines to what extent breeding for herbivore-resistant plant varieties and the application of selected natural enemies as biocontrol can additively contribute to reduce pest levels^6,11^. Maximizing synergies between the strategies of host plant resistance and biological control could help to meet our demand for sustainable and more environmentally friendly crop protection^12,13^. Therefore, it is important to identify plant genotypes which naturally represent low food quality for the herbivore (i.e. strong direct defence) and inversely improve the parasitoid fitness (i.e. facilitating biological control). However, in the process of breeding and assessing resistant crop varieties, their effect on the performance of biological control agents is scarcely considered^13,14^. Thus, little is known about the extent of potential (in)compatibilities of biocontrol and current plant breeding programs.

Parasitoids, which are widely used as biocontrol agents, are especially sensitive to plant metabolites consumed by herbivores. Unlike insect predators, which consume many different prey items to fulfil their nutritional needs, parasitoid progeny are confined to resources provided by a single herbivore host^8,15^. It has been previously shown that dietary quality of the food plant can influence composition of the herbivore’s haemolymph, and as such, the quality and quantity of available resources for the parasitoid^5,13,16–19^. Given the wide variety and function of chemical compounds within plant tissues, host plant dietary quality is hence a complex property, and its assessment requires a multifaceted approach^1^. However, most previous research involving direct plant defences and parasitoids has been limited to the role of acutely toxic secondary plant compounds^13,20,21^, which are often highly specific to certain plant families. A one-sided focus on toxic, plant family-specific secondary compounds limits the possibility of drawing general conclusions because such findings are difficult to extend to other plant families. Other less or non-toxic secondary  metabolites as well as primary ones have generally received less attention, and have usually been studied in isolation of each other^5,18,20^.

In this study we focused on wild woodland strawberry (*Fragaria vesca* L.), a promising resource for anti-herbivore resistance which can contribute to breeding programs for the commercially cultivated garden strawberry (*F*. x *ananassa*)^22–25^. Previously, we found very high intraspecific variation in plant resistance against the strawberry leaf beetle (*Galerucella tenella* L., Coleoptera: Chrysomelidae) among wild woodland strawberry genotypes^25^. In order to better estimate whether breeding for resistance would affect parasitoid performance in our system it is necessary to quantify parasitoid performance along a gradient of low to high plant resistance to the herbivore host.

From the wild woodland strawberry genotypes previously screened for herbivore performance^25^, we selected eight resistant and eight susceptible genotypes to investigate the effects of actual phenotypic resistance on their potential to facilitate biological control. We examined the variability in development and survival of the larval endoparasitoid species *Asecodes parviclava* Thompson (Hymenoptera: Eulophidae) in relation to diet source (i.e. plant genotype) and plant resistance level. Additionally, we assessed the chemical variation of the selected woodland strawberry genotypes to determine the bottom-up influence of plant chemistry on the outcome of parasitism. We subjected the leaves of the plant genotypes to non-targeted GC/TOF-MS (gas chromatography/time of-flight-mass spectrometry)-based metabolite profiling. The range of primary and secondary metabolites quantified from the profiling was then tested for its association with the success or failure of the parasitism per plant genotype.

Specifically, this study tested the following questions: 1) how are performance and survival of a parasitoid influenced by plant-resistance to the herbivores?; and 2) which primary and secondary plant metabolites contribute to the successful development of the associated parasitoid species *A. parviclava*.?

## Results

### Effect of plant genotype and resistance on the outcome of parasitism

**Figure 1:**
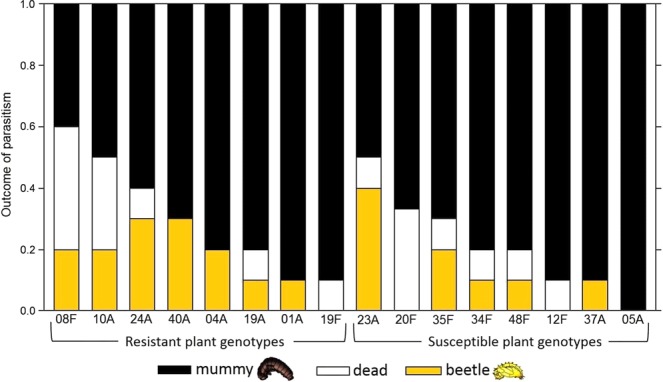
The effect of plant genotype on the outcome of parasitism. Proportion of parasitized beetle larvae that became a parasitoid mummy (black), died pre-pupa (white) or a beetle pupa (yellow) across 16 wild woodland strawberry genotypes on which the beetle larvae fed. Note that in the figure, woodland strawberry genotypes are presented according their resistance level against strawberry leaf beetle although plant anti-herbivore resistance did not affect the outcome of the parasitism. *P* < 0.05 based on a multinomial logistic regression.

The genotype of the plant material consumed by the parasitized beetle larvae had a significant effect on the success and outcome of the parasitism (χ^2^ = 45.159; df = 30; *p* = 0.0373; Fig. 1). The mummification rate (parasitized larva developed into a mummy) ranged from 40% to 100% depending on the plant genotype the larva had been feeding on (Fig. 1). However, plant resistance against the strawberry leaf beetle (plant genotype categorized as resistant or susceptible) had no significant effect on the outcome of the parasitism (χ^2^ = 1.617; df = 2; *p* = 0.4455).

The parasitoid mummies had an average of 2.9 (and maximum of 5) parasitoid pupae per mummy. Neither plant resistance level nor plant genotype had a significant effect on the number of pupae inside the mummies. However, the developmental time from parasitism to mummy formation had a highly significant influence on the number of pupae, with shorter time associated with a higher number of pupae (F^1, 90^ = 18.41, *p* < 0.001).

### Primary and secondary plant metabolites contributing to parasitoid development and plant resistance against Galerucella tenella

Based on GC/TOF-MS profiling of woodland strawberry leaves, a total of 81 compounds were detected and quantified. From the observed molecular features, 51 could be annotated (i.e., recognized by mass spectrum and retention index), 15 were structure-classified and 15 non-identified mass spectral tags (see Supplementary Table 3). Besides several carbohydrates, a range of amino acids, fatty acids, miscellaneous organic acids, as well phenolics and terpenoids constituted the compound profile of woodland strawberry leaves.

**Figure 2:**
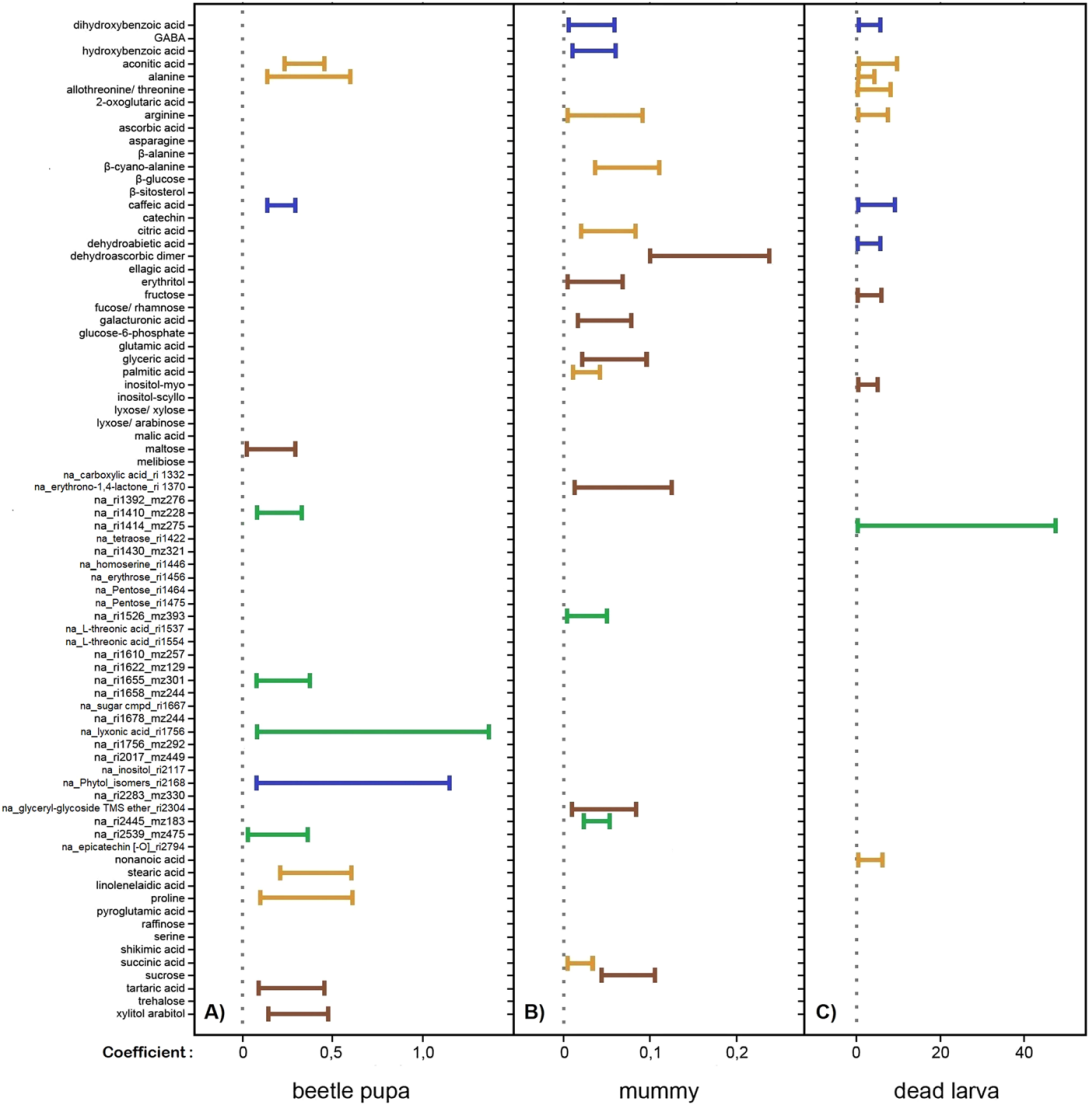
Coefficient plot of plant metabolites and parasitism outcome. Results of partial least square generalised linear model (PLS GLM) analysis evaluating the association of woodland strawberry leaf compounds from the metabolite profiles (81 identified metabolites and non-identified mass spectral tags) and parasitized beetle larvae that A) developed successfully to a beetle pupa B) became a parasitoid mummy, or C) died in the pre-pupal stage (mean ± 95% bootstrapped confidence intervals (CI)). Only the CIs of significant and positive associations are depicted to increase readability. The colour of the CIs indicates the plant compound group including carbohydrates (dark brown) and amino acids, lipids, organic acids (light brown), secondary compounds (blue) as well as non-identified compounds (green). Coefficients and CIs establishing compound significance derive from the non-adjusted models. See Supplementary Table 3 for indication of compound significant following adjustment made for multiple tests.

**Figure 3:**
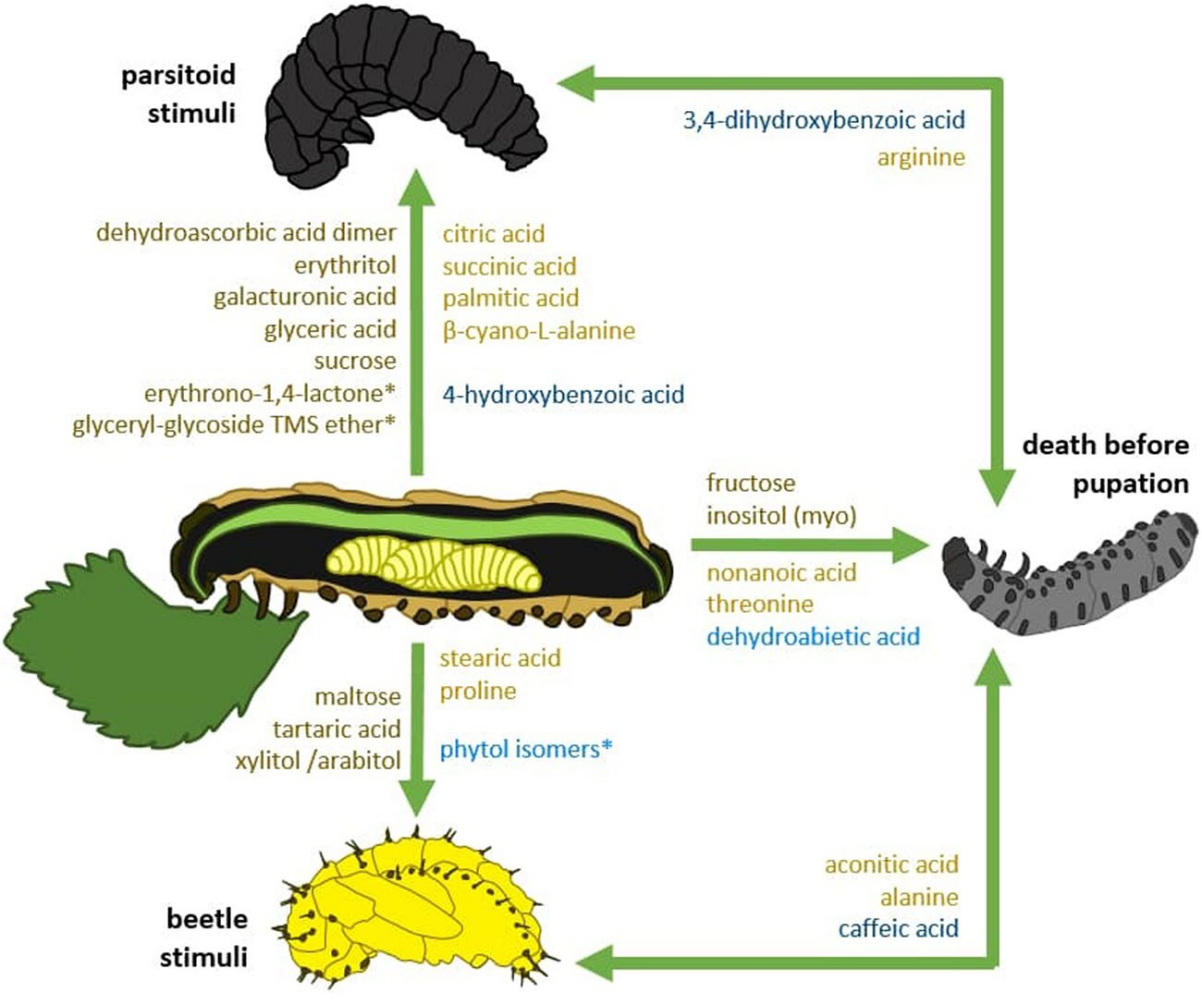
Schematic of the key plant compounds associated with the outcomes of the parasitism. The green arrows indicate plant compounds correlated to one or to two outcomes. The plant compounds consist of primary compounds including carbohydrates (dark brown) and amino acids, lipids, organic acids (light brown) as well as secondary compounds including phenolics (dark blue) and terpenoids (light blue). Illustration by Daniela Weber.

Partial least squares regression successfully identified plant compounds associated with the outcome of parasitism (host larvae died; larvae became a beetle pupa; larvae became a mummy) (Fig. 2). This analysis revealed that 39 compounds had a significant positive association with at least one of the three possible outcomes (27 compounds retained significance after adjustment for multiple tests, Supplementary Table 3). The successful formation of a mummy was associated by the presence of 16 compounds, with carbohydrates and unidentified compounds being the most influential chemical groups (Figs. 2 and 3). Resistance against parasitism was positively associated with the presence of 14 compounds mainly from the group of unidentified metabolites (Figs. 2 and 3). The pre-pupal mortality of beetles was significantly associated with 11 compounds from different compound groups (Figs. 2 and 3).

Although most compounds were significant only for one specific type of the three possible outcomes, a small number of them  were significant for two outcomes. The amino acid arginine and the phenolic compound 3,4-diydroxybenzoid acid were positively associated with both pre-pupal mortality as well as a successful parasitism, whereas aconitic acid, alanine, and caffeic acid (citric, amino, and phenolic acids, respectively) were positively  associated with pre-pupal mortality but also resistance against parasitism (Figs. 2 and 3).

In the absence of parasitism, partial least squares regression identified a total of 27 plant compounds associated with plant resistance and/or susceptibility to *G. tenella*  (the host of the parasitoid). Plant resistance was associated with 18 compounds whereas plant susceptibility was associated with nine compounds (Table 1; Supplementary Table 3). In cross-referencing the set of 18 compounds associated with plant resistance in the absence of parasitism with the 26 compounds associated with herbivore mortality when parasitism was present (i.e., cases where mummy development was successful, or pre-pupal mortality occurred for both host and parasitoid larva), only five compounds were identified as overlapping (Supplementary Table 3). Whereas in the case of cross-referencing the set of nine compounds associated with plant susceptibility in the absence of parasitism with the 13 compounds associated with the herbivore resistance to parasitism, only a single compound (alanine) overlapped.

**Table 1:** Coefficient table of plant compounds related to woodland strawberry resistance.

Compound	Coefficient	CI lower	CI upper
***Resistance***			
dihydroroxybenzoic acid	10.003	0.842	124.359
hydroxybenzoic acid	7.174	0.514	429.221
2-oxoglutaric acid	1.826	0.462	21.021
β-alanine	2.299	0.3	25.397
catechin	3.092	0.574	24.225
palmitic acid	2.726	0.581	76.57
inositol-scyllo	17.419	2.027	131.412
na_ri1392_mz276	2.173	0.161	16.894
na_ri1430_mz321	1.002	0.056	46.783
na_ri1526_mz393	1.662	0.361	54.147
na_ri1756_mz292	1.066	0.287	20.696
na_ri2017_mz449	4.954	0.792	107.88
na_ri2539_mz475	0.974	0.108	65.895
na_ri2794_mz280	2.235	0.217	33.328
stearic acid	1.079	0.059	13.8
proline	2.92	0.34	52.136
shikimic acid	2.543	0.701	87.043
tartaric acid	14.972	0.95	1435.536
***Susceptibility***			
alanine	−5.967	−69.162	−1.66
arginine	−1.952	−10.012	−0.223
ascorbic acid	−2.412	−13.686	−0.378
asparagine	−2.517	−521.105	−0.502
β-glucose	−6.024	−337.384	−0.875
β-sitosterol	−2.365	−36.622	−0.334
ellagic acid	−4.954	−95.122	−0.585
malic acid	−2.243	−7.021	−0.338
na_ri1446_mz218	−1.503	−13.67	−0.17

Results of XX analysis testing the association of woodland strawberry leaf compounds from the metabolite profiles (81 identified metabolites and non-identified mass spectral tags) and plant resistance previously screened as antibiosis and antixenosis against *G. tenella*^25^ (mean ± 95% bootstrapped confidence intervals (CI)). Positive CIs represent an association between the compounds and plant being resistant while negative CIs represent an association between the compounds and plant being susceptible. Only significant associations are given, full table can be found from Supplementary Table 3.

## Discussion

This study shows that the effects of plant nutritional quality of wild strawberry  on parasitoid performance were not primarily based on direct plant resistance to the insect herbivore, but on the overall effect of primary and secondary plant metabolites on the quality of the herbivore as a parasitoid host. This finding indicates that plant quality, in terms of both primary and secondary chemical composition, plays an important role in shaping tritrophic interactions. The underlying mechanisms were not identified in this study, but one may speculate that some plant metabolites could  compromise host immune responses^26,27^, while other compounds could  have direct antagonistic effects on one or both of the interacting insects. Interestingly, plant traits that confer resistance against the herbivore do not necessarily affect the parasitoid negatively. By contrast, some plant resistance compounds, e.g. palmitic acid and hydroxybenzoic acid, even contributed to successful parasitism. The survival rate of the parasitoid species *Asecodes parviclava*, although depending on plant genotype, was not congruent with the anti-herbivore resistance among the selected woodland strawberry genotypes. From a plant protection perspective, this shows that even plant genotypes with high resistance against herbivores can facilitate successful biocontrol.

While survival of immature parasitoids inside the beetle host varied between plant genotypes, parasitoid development time and weight of the mummy did not differ significantly between plant genotypes. This indicates that developmental time and mummy weight are more conserved life history traits in the parasitoid and certain thresholds must be reached for completing the larval stage.

Although primarily studied separately (but see e.g. van Geem *et al*., 2016^28^), our study examined the combination of primary and secondary plant metabolites, and how each detected compound potentially correlated with parasitism outcome. From the eighty-one compounds detected in the tested plant material, we found that forty compounds were significantly associated with herbivore or parasitoid survival. Five compounds were significantly associated with pre-pupal mortality of the host larvae and also either with parasitoid survival (two compounds) or beetle survival (three compounds). No overlap in compound association occurred between successful parasitism (i.e. parasitoid survival) and herbivore resistance against parasitism (i.e. beetle survival), indicating that the two interacting insects are affected by different phytochemical traits.

For primary metabolites, we found amino acids, a range of carbohydrates, few lipids and organic acids connected to the citric acid cycle in the leaf material. Amino acids are essential for the development of insects, although requirements differ with respect to insect species. Conversely, in some cases, individual amino acids can also show toxic effects for herbivores^29,30^. In our study, parasitoid performance correlated positively with β-cyano-L-alanine and arginine, with the later previously found to be an essential requirement for rearing different parasitoid species in artificial media^31^. Interestingly, threonine, which is also listed as an essential amino acid for artificial parasitoid rearing, was associated with pre-pupal mortality of the host larva in our system. In contrast, proline and alanine appeared to impair parasitoid  development. Alanine was not only associated with the increased beetle survival but also with pre-pupal mortality of the host larva. This amino acid seems to be harmful foremost for the parasitoid species *A. parviclava*, whereas the beetle appears to be less sensitive to alanine. Alanine is mentioned to increase anti-herbivore resistance to a grass moth in sorghum^32^, yet also to act in the combination with sucrose as feeding stimulant in another strawberry leaf beetle *Galerucella vittaticollis* (Baly)^33^. Although seemingly contradictory, previous studies have shown that sucrose and other plant sugars can act as effective masking agents to repellent and/or harmful plant compounds^34,35^ which can stimulate the consumption of material containing harmful compounds otherwise avoided by the herbivore.

Another sugar, found in our profiling, myo-inositol, has been also previously shown to function as a potent masking agent for noxious compound such as caffeine or aristolochic acid^35^ and was in our study associated with increased pre-pupal mortality of the host larva. However, most identified carbohydrates (dehydroascorbic acid dimer, erythritol, galacturonic acid, glyceric acid, sucrose) correlated positively with parasitoid performance. These findings suggest that carbohydrates are important plant resources for the parasitoid species *A. parviclava* enabling its successful development.

Apart from nonanoic acid, a potent antifeedant against pine weevils^36^, and which was here found to correlate with pre-pupal mortality of the host larva, a direct uptake of lipids from the leaf material appear to play a minor role for parasitoid fitness. Besides representing a direct energy source, carbohydrates can be converted into lipids. A diet rich in carbohydrates generally stimulates the accumulation of lipid reserves^37^, which seems to serve the needs of the parasitoid species *A. parviclava* better than the direct lipid uptake from the plant. Most parasitoid species consume host lipids instead of synthesizing them *de novo*, and often manipulate the host’s metabolism in their own favor, ensuring a sufficient lipid supply during development^38^.

Other primary compounds found in the leaves are connected to the citric acid cycle and seemingly (i.e. positively correlated) beneficial for the parasitoid with the exception for aconitic acid. We found, that aconitic acid correlated with the host aversion of parasitism as well as mortality of the pre-pupal host larva.

Of the secondary compounds found in the leaves, phenolics mainly correlated with parasitoid survival, whereas caffeic acid was positively linked to beetle survival and pre-pupal mortality of the host larva. Caffeic acid is a pro-oxidant in the herbivore’s gut, but may also cross the gut wall and be found in unbound form in the haemolymph 24 h after ingestion^39,40^. Caffeic acid has also been shown to exhibit pro-oxidant tendencies in the haemolymph, especially after longer periods of time^40^. Surrounded by the hemolymph, the parasitoid larvae would be directly exposed to harmful oxidative stress that can be caused by the pro-oxidant. It appears that although caffeic acid might negatively affect also the beetle, it seems to impair mainly the parasitoid species *A. parviclava*.

The detected terpenoids on the other hand, seem to impair the development of the parasitoid species *A. parviclava*. Dehydroabietic acid was correlated to increased general larval mortality, and phytol appeared to increase beetle survival. Dehydroabietic acid has been previously demonstrated to function as an insect juvenile hormone antagonist^41^, and represents a strong indicator of weevil resistance in pines^42^.

To sum up, we can conclude that both primary and secondary plant metabolites seem to be equally important for the larval development of the parasitoid species *A. parviclava*. Plant tissues contain a wide variety of chemical compounds and plants usually employ multiple lines of defence concurrently, rather than relying on individual defensive mechanisms^1,6^. Thus, in order to gain a better, more holistic understanding of the impact that plant anti-herbivore resistance has on the development of parasitoids, a wider range of plant compounds need to be taken in account. These results suggest that studying only a small set of either primary or secondary metabolites, putative interactions – synergistic as well as antagonistic – between plants and parasitoids (as well as between the compounds themselves) are likely to remain unnoticed.

Although we have screened a broad range of plant compounds, including both primary and secondary metabolites, it is important to note that our profiling is not complete. Even more plant compounds (which were not included in our study) are probably significant for tritrophic interactions. However, this study forms a very important and novel platform for the development of a strategic breeding program for optimising strawberry for biological control.

Plant-mediated parasitoid performance (growth, survival) can be expected to impact parasitism rate and thus play an important role shaping community structures. Previous studies have found that parasitism rates of the parasitoid species *A. parviclava* varied strongly between wild plant populations^26,43^. Spatial variation in parasitism may of course depend on many different underlying factors^1^. However, taking our findings into account, intraspecific variation in plant quality may contribute to previously reported variations in parasitism of *Galerucella*^26,43,44^ which underpins the importance of including plant quality when studying plant-herbivore-parasitoid interactions.

From an applied perspective, our study shows that it should be possible to breed woodland strawberry for enhanced plant resistance without impairing the biological control function of the parasitoid. Several of the wild woodland strawberry genotypes with high beetle resistance were able to optimally sustain the parasitoid. Thus, integrated pest management can benefit from evaluating novel crop varieties from a biocontrol perspective, and when possible, by removing undesired plant resistance traits that interfere with biocontrol from future breeding programs^45^. Moreover, an efficient biocontrol can contribute to prevent or decelerate herbivore adaptation to plant resistance^13,46^. Thus, simultaneously optimizing plants both for resistance and biocontrol would increase cropping security and reduce pesticide dependence.

## Materials and Methods

### Study plant and plant material

The woodland strawberry (*Fragaria vesca* L., Rosaceae) is an herbaceous perennial plant occurring throughout most of the Holarctic. It grows naturally in various half shaded and sunny habitats such as open forests and edges of farmland^47,48^. Across our study area (central Sweden) flowers are produced throughout the entire growing season from May until September, with a clear peak in early June^48^. In addition to sexual reproduction, woodland strawberry is also capable of clonal reproduction and forms a high number of runners that grow quickly into self-sustaining plants^47^.

The 16 plant genotypes used in this study were derived from distinct wild populations in Uppsala County, Sweden, during spring 2012^24,25,49^ (Supplementary Material Table 1). The entire collection consists of 86 plant genotypes that have been screened for resistance against the strawberry leaf beetle^25^. For this study we selected eight plant genotypes with high resistance and eight genotypes with high susceptibility against strawberry leaf beetles to include the contrasting ends of the resistance spectra for this experiment^25^.

Following the clonal propagation for several generations, forty runners per plant genotype were transplanted into a common garden close to Uppsala (N59.741°, E17.684°) in autumn 2013. The common garden was covered with fabric mulch (Weibulls Horto) as a weed barrier, and no irrigation, herbicides or fertilizers were used. The full protocol for the common garden establishment is available in Muola *et al*.^49^.

### Study insects

The insect herbivore and the parasitoid used in this study are native to Fennoscandia and sympatric in their northern distribution. The strawberry leaf beetle *Galerucella tenella* L. (Coleoptera: Chrysomelidae; Fig. 2) is oligophagous on several species of the Rosaceae family, including a range of *Fragaria* spp., and is commonly found on meadowsweet (*Filipendula ulmaria* (L.) Maxim.)^50,51^. It can reach pest status especially in organic strawberry cultivations in the Nordic countries^19,52^, the Baltic States^53^, and Russia^54^. The strawberry leaf beetle is a univoltine species that hibernates as adult in the topsoil before emerging during April to May when the plants start to grow after winter. By the end of June, the mated female beetles oviposit directly on the plant they feed on and the larvae hatch after approximately two weeks depending on the climatic conditions^51^. Like the adult beetles, the larvae feed on the leaves and flowers for two to four weeks until they pupate in the upper soil layer^51^.

**Figure 4:**
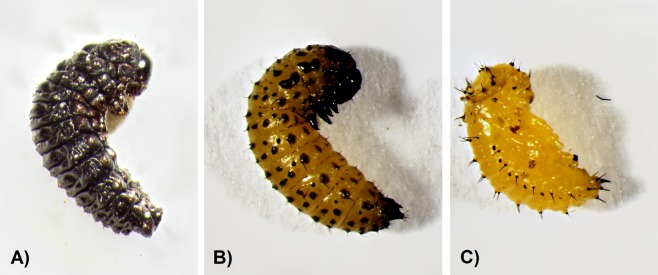
Photographs of (**A**) a mummified larva of the strawberry leaf beetle (*Galerucella tenella*) inclosing pupae of the parasitoid species *Asecodes parviclava* (**B**) a pre-pupal stage before the beetle larva turns into a parasitized mummy or beetle pupa (**C**) a beetle pupa of the strawberry leaf beetle. Photos by Daniela Weber.

*Asecodes parviclava* Thompson (Hymenoptera: Eulophidae) is the only known parasitoid species of *G. tenella* in Sweden. This gregarious endoparasitoid species attacks *G. tenella* in the early larval stage, inserting one or more eggs into the body of the beetle larvae. The parasitized beetle larva continues to feed and grow until the last stages of parasitism, when parasitoid larvae prepare for pupation and eventually consume all the tissue of their insect host from within. The remaining beetle cuticle turns into a mummified black shell (thereafter mummy, Fig. 4) around the pupating parasitoids. Most parasitoids overwinter in their mummy as pupae and emerge as adults during the next summer, when new beetle larvae are again available^50,55^.

### Insect rearing

Adult *Asecodes parviclava* were reared from naturally occurring larvae of the strawberry leaf beetle, which were collected from natural meadowsweet stands in the Skeppsvik Archipelago, Sweden (N63.779°, E20.616°; N63.762°, E20.594°) in July 2015. The mummies were placed individually in 1.5 mL micro tubes, sealed with a piece of cotton wool and stored over winter at a shaded and sheltered location outdoors at SLU Ultuna campus. In late spring, with rising temperatures, the tubes were monitored daily for emerging adult parasitoids. Upon emergence, the parasitoids were transferred to 250 ml plastic containers with a feeding station (i.e. a piece of cotton providing diluted honey) and allowed to mate freely.

To obtain beetle larvae for parasitism, we collected mating couples of adult *G. tenella* during May 2016 from six distinct natural *Filipendula ulmaria* populations in the area around Uppsala (N59.809°, E17.667°; N59.788°, E17.664°; N59.806°, E17.652°; N59.806°, E17.665°; N59.782°, E17.753°; N59.833°, E17.914°). The collected individuals were randomly mixed and placed in meshed cages containing a mix of woodland strawberry chosen randomly among the plant genotypes available in the common garden. The cages were kept in a greenhouse (15 °C, LD 16:8 h photoperiod, 80% RH) and after a 24 h oviposition period, plants with eggs were retrieved and placed in a separate cage to allow the development of the eggs into larvae. The larvae used for experiments were in the second instar and of approximately the same size.

### Experimental setup

Larvae of the leaf beetles reared on resistant or susceptible plants were used to study effects of plant resistance and quality on parasitoid performance. Second instar beetle larvae were presented individually to a mated *A. parviclava* female in a 1.5 mL micro tube. The beetle larvae were considered parasitized when the parasitoid was observed to have inserted its ovipositor for at least 10 seconds and showed no further interest in the larva. This method to experimentally parasitize *Galerucella* beetles normally results in close-to 100% successful oviposition (Stenberg, *pers. obs*.), but we did not dissect the experimental larvae used for this study to confirm that all of them contained parasitoid eggs. Each parasitized larva was randomly assigned to feed one of the sixteen plant genotypes and transferred to a 30 ml plastic container containing one detached, middle aged leaf of the given plant genotype. Ten parasitized beetle larvae were individually fed plant material from each of the 16 plant genotypes, resulting in 160 rearing containers. The rearing containers were kept in a climate chamber (15 °C, LD 16:8 h photoperiod, 80% RH). We checked the parasitized larvae daily and replaced the leaves every third day. Upon pupation, we noted whether the parasitism was successful (i.e. the host larva turned into a mummy) or not (i.e. the host larva died or turned into a beetle pupa). For each mummy we recorded the date of occurrence and the weight three days after it reached the pupal/mummy stage. The development time of parasitoids was measured as the number of days between parasitism and mummification. The mummies were transferred to 1.5 ml Eppendorf tubes sealed with cotton wool and checked daily for adult egression. At the end of August the tubes with the mummies were transferred outdoors at the SLU campus in Ultuna to be stored over winter. In June the following year, we counted the enclosed parasitoid pupae by carefully dissecting the mummies under a microscope.

### Metabolomic analysis of the tritrophic effects of food plant quality

To investigate which phytochemical compounds correlate with plant resistance to *Galerucella tenella*, and to verify whether the outcome of the larval development, following parasitism, is influenced by the general leaf chemistry, we conducted metabolomics profiling of leaf tissue from 14 of the 16 plant genotypes used in the experiment (two genotypes were left out by mistake). Leaf samples (of the same age, quality, and location as used in the parasitoid experiment) were sampled from strawberry leaf beetle damaged plants in the common garden. Two clonal plants from each genotype were separately sampled for metabolic profiling, from which five leaves per plant were collected and pooled. Compound concentrations representative of each genotype were then generated by taking the mean compound value of these two clonal replicates. Within 2–3 hours, samples were stored initially at −20 °C, and then at −80 °C before freeze drying. The dried leaves were grinded to a fine powder using an electric mill (IKA A 10 basic model) and stored in the dark to avoid degradation by extended exposure to sunlight.

Metabolomic profiling of the leaf tissue by GC/TOF-MS was performed at the Swedish Metabolomics Centre, Umeå, Sweden. For the extraction a combined extraction buffer (Chloroform/Water/Methanol (20/20/60, v/v)) was used and the procedure of sample preparation, derivatization, GC/TOF-MS analysis was carried out according to A *et al*.^56^. A detailed description of the procedure as well as all involved chemicals and standards can be found in the Supplementary Material. All non-processed GC/TOF-MS files were pre-treated using custom scripts (base-line correction, chromatogram alignment, data compression, Multivariate Curve Resolution) and subsequently identified by comparisons with libraries of retention time indices and mass spectra.

### Statistical analysis

We used the multinomial logistic regression function ‘multinom’ in the R package ‘nnet’^57^ to estimate the probability for the occurrence of each potential outcome of the parasitism (host larva died; larva became a beetle pupa; larva became a mummy) in relation to the two plant resistance levels i.e. susceptible and resistant (model 1) and in relation to each of the 16 plant genotypes selected for this study (model 2). These terms were ran in separate models due to the fact that this function was unable to handle nested structures.

We used a linear model to assess variation in the total number of pupae per mummy among the 16 plant genotypes. We set the total number of pupae per mummy as the response variable and included weight of the mummy, development time from parasitism until mummy formation, and plant genotype nested in plant resistance level (susceptible or resistant) in the model. Plant genotype was treated here as a fixed factor since the used genotypes were not chosen randomly but were carefully selected based on the herbivore resistance level from a larger set of genotypes previously studied in Weber *et al*.^25^. A significant interaction term between weight and developmental time was retained in the final model, whereas all other interactions were excluded due to insignificance. For model validation, we assessed normality of the residuals by visual examination and conducted a Levene’s test to check for equality of variances.

Partial least squares generalised linear models (PLS GLMs) were used on the GC/TOF-MS data to test whether the respective outcome of the parasitism can be predicted based on individual compounds in the leaves of the plant genotypes on which the insect host had been feeding. PLS GLMs are considered a type of hybrid model that incorporates the use of both principal component analysis (PCA) and generalized linear regression. As such, these models harness the relative advantages of each^58^. Perhaps most critically, valid inferences can be made based on a large number of highly inter-correlated predictor variables, even when this far surpasses the number of response cases. Here, for instance, 14 parasitism response cases were regressed on 78 compound predictor variables. And secondly, binomial or Poisson error distributions can be explicitly accommodated.

We used the R package “plsRglm”^58^ to fit three Poisson PLS GLMs. These models were fitted with compound concentration as predictor variables, and a count of each parasitism outcome as an individual response per model (i.e., the ‘number of host larva died or not’, ‘number of larva that became a mummy or not’, or ‘number of beetles developed to pupa or not’). While currently no means exist to extend PLS GLMs for multinomial responses, our approach nonetheless could still account for dependency between parasitism responses. This was due to the fact that parasitism outcomes were mutually exclusive; i.e., a significant positive association between a compound and a response in one model (e.g., number of beetles developed), by default indicated a significant negative association in at least one other (e.g., number of larva that became a mummy). Only positive associations were deemed to be informative, as negative associations provided only redundant information. These *a priori* assumptions indicate that the need to adjust for multiple tests may arguably be relaxed. However, we nonetheless present significance results for both normal and adjusted tests (Supplementary Table 3). The adjustment was implemented using the ‘confints.bootpls’ function of the plsRglm package, where a Bonferroni type correction was applied to the alpha level for significance when estimating coefficient CIs.

For each of the three PLS GLMs, we used an AIC information criteria approach to select of the optimal number of components that should be fit in order to minimize AIC (i.e. three for the ‘host larva died’ and ‘larva became a mummy’ models, and four for the ‘beetle pupa’ model). For each compound in each model, 95% confidence intervals (CIs) were estimated based on 1000 parametric bootstraps. A significant association between the compound and the outcome was hence indicated where the range of the CI did not include zero. For model validation, we tested for over-dispersion using the ratio of the residual deviance and the residual’s degrees of freedom.

A PLS GLM was also run to test the link between the 78 compound predictor variables and plant resistance/susceptibly, encoded as a binary response. This model was fitted using a binomial family link function, but otherwise all analysis and validation procedures followed as for the Poisson models described above.

All statistical analyses were run using R v.3.5.1 and RStudio v. 1.1.456.

## Supplementary information

Supplementary information.
