# Supplementary material for: Plant resistance does not compromise parasitoid-based biocontrol of a strawberry pest
Source: Sci Rep. 2020 Apr 3;10:5899. doi: 10.1038/s41598-020-62698-1 (PMC7125231; doi:10.1038/s41598-020-62698-1)
Supplement: Supplementary file 1 — Supplementary information. [file 41598_2020_62698_MOESM1_ESM.pdf]

Plant resistance does not compromise parasitoid-based biocontrol of a strawberry pest

Daniela Weber[1,*], Paul A. Egan[1], Anne Muola[1,2], Lars E. Ericson[3] & Johan A. Stenberg[1]

[1]Department of Plant Protection Biology, Swedish University of Agricultural Sciences, Box 102, 23053 Alnarp, Sweden

[2]Biodiversity Unit, University of Turku, 20014 Turku, Finland

[3]Department of Ecology and Environmental Science, Umeå University, 90187 Umeå, Sweden

Corresponding author: Daniela Weber, Sundsvägen 14, SE- 230 53 Alnarp, Sweden
daniela.weber@slu.se

Supplementary Table 1. *Collection site coordinates of woodland strawberry genotypes.*

The 16 woodland strawberry (*Fragaria vesca*) genotypes used in the study were selected from a collection of 86 wild woodland strawberry genotypes derived from geographically distinct locations across Uppsala County, Sweden, in spring 2012 (Egan et al., 2018; Weber et al., 2019). The division of the plant genotypes to susceptible and resistant is based on the antibiosis level against the strawberry leaf beetle, *Galerucella tenella* (Weber et al., 2019). Coordinates are given in decimal degree format and based on the international WGS 84 (World Geodetic System).

Plant resistance level	Plant genotype	Longitude	Latitude
Susceptible	01 A	17.4607833	60.58625
	04 A	17.4682	60.4927166
	08 F	18.5696667	60.3076166
	10 A	17.5596667	60.3738
	19 A	18.2141	60.1582667
	19 F	18.1865667	60.1976166
	24 A	17.65225	60.0747
	40 A	18.0445	59.7585834
Resistant	05 A	17.6124333	60.4346833
	12 F	18.0151833	60.304
	20 F	18.3433333	60.1018
	23 A	17.4720834	60.1559666
	34 F	18.2901	59.88625
	35 F	16.8041	59.9318167
	37 A	17.3795834	59.9105334
	48 F	17.3085166	59.6393334

GC-MS based metabolomics profiling of food plant quality

Metabolomic profiling of the leaf tissue by GC-MS (gas chromatography/time of-flight-mass spectrometry) was performed at the Swedish Metabolomics Centre, Umeå, Sweden. The sample preparation, derivatization and GC-MS analysis was carried out according to [1] and the used reagents, solvents, standards and references can be found in Supplementary Table 2.

For the sample preparation we weighed 2 mg aliquots of the powered leaf material in fresh 1.5 ml centrifuge tubes and added 1000 µL of extraction buffer (Chloroform/Water/Methanol (20/20/60, v/v)) including internal standards for GC-MS. Each sample was shaken at 30 Hz for 3 minutes in a mixer mill and proteins were precipitated at +4 °C on ice. After 10min in a centrifuge at +4 °C, 14 000 rpm, 50 µL of each supernatant was transferred to micro vials and evaporated to dryness in a speed-vac concentrator. A quality control pool was made by combining 50µL extract from all samples and 200 µL form the pool was run as quality control throughout the GCMS analysis.

The samples were analysed according a designed randomized run order on the GC-MS. In detail 1 µl of the derivatized sample was injected in splitless mode by a CTC Combi Pal auto sampler (CTC Analytics AG, Switzerland) into an Agilent 6890 gas chromatograph equipped with a 10 m x 0.18 mm fused silica capillary column with a chemically bonded 0.18 µm DB 5-MS UI stationary phase (J&W Scientific).

The injector temperature was 270 °C, the purge flow rate was 20 mL min-1 and the purge was turned on after 60 seconds. The gas flow rate through the column was 1 mL min-1, the column temperature was held at 70 °C for 2 minutes, then increased by 40 °C min-1 to 320 °C, and held there for 2 minutes. The column effluent was introduced into the ion source of a Pegasus III time-of-flight mass spectrometer, GC/TOFMS (Leco Corp., St Joseph, MI, USA). The transfer line and the ion source temperatures were 250 °C and 200 °C, respectively. Ions were generated by a 70 eV electron beam at an ionization current of 2.0 mA, and 30 spectra s-1 were recorded in the mass range m/z 50 - 800. The acceleration voltage was turned on after a solvent delay of 150 seconds. The detector voltage was 1500-2000 V.

For the GC-MS data, all non-processed MS-files from the metabolomic analysis were exported from the ChromaTOF software in NetCDF format to MATLAB R2016a (Mathworks,

Natick, MA, USA), where all data pre-treatment procedures, such as base-line correction, chromatogram alignment, data compression and Multivariate Curve Resolution were performed using custom scripts. The extracted mass spectra were identified by comparisons of their retention index and mass spectra with libraries of retention time indices and mass spectra [2]. Mass spectra and retention index comparison was performed using NIST MS 2.0 software. Annotation of mass spectra was based on reverse and forward searches in the library. Masses and ratio between masses indicative for a derivatised metabolite were especially notified. If the mass spectrum according to SMC's experience was with highest probability indicative of a metabolite and the retention index between the sample and library for the suggested metabolite was ± 5 (usually less than 3) the deconvoluted "peak" was annotated as an identification of a metabolite.

Supplementary Table 2. *Chemicals used in the metabolomics profiling.*

Reagents, solvents, standards, reference as well as tuning and stable isotopes internal standards used in sample preparation, derivatization and GC-MS analysis for the metabolomics profiling of 16 woodland strawberry (*Fragaria vesca*) genotypes.

Solvent	Description
Methanol	HPLC-grade,Fischer Scientific (Waltham, MA, USA)
Chloroform<br>Acetonitrile<br>2-Propanol<br>H_2O, Milli-Q	Suprasolv for GC, Merck (Darmstadt, Germany)<br>HPLC-grade, Fischer Scientific (Waltham, MA, USA)<br>HPLC-grade, VWR (Radnor, PA, USA)
Reference and tuning standards	Description
Purine<br>HP-0921	4 µM, Agilent Technologies (Santa Clara, CA, USA)<br>Hexakis(1H, 1H, 3H-tetrafluoropropoxy) phosphazine<br>1 µM, Agilent Technologies (Santa Clara, CA, USA)
Calibrant	Description
ESI-TOF<br>HP-0321	ESI-L Low Concentration Tuning Mix, Agilent Technologies (Santa Clara, CA, USA)<br>(Hexamethoxyphosphazine), 0.1 mM, Agilent Technologies (Santa Clara, CA, USA)
Stable isotopes internal standards	Desription,
L-proline-13C5<br>alpha-ketoglutarate-13C4<br>myristic acid-13C3<br>cholesterol-D7<br>Succinic acid-D4,<br>salicylic acid-D6<br>L-glutamic acid-13C5,15N<br>putrescine-D4<br>hexadecanoic acid-13C4<br>D-glucose-13C6<br>D-sucrose-13C12	Cil (Andover, MA, USA)<br>Cil (Andover, MA, USA)<br>Cil (Andover, MA, USA)<br>Cil (Andover, MA, USA)<br>Sigma (St. Louis, MO, USA)<br>Sigma (St. Louis, MO, USA)<br>Sigma (St. Louis, MO, USA)<br>Sigma (St. Louis, MO, USA)<br>Sigma (St. Louis, MO, USA)<br>Sigma (St. Louis, MO, USA)<br>Sigma (St. Louis, MO, USA)

Supplementary Table 3. *Plant metabolites tentatively identified in leaf samples collected from 14 woodland strawberry genotypes based on GC/TOF-MS analysis.*

Total of 81 compounds were detected through the GC/TOF-MS profiling from the leaf samples that were derived from 14 of the 16 woodland strawberry (*Fragaria vesca*) genotypes used in the parasitism experiment. 51 from the observed compounds could be recognized by their mass spectrum and retention index, 15 were structure-classified and 15 non-identified mass spectral tags. Besides phenolics and terpenoids, the compound profile of the woodland strawberry leaves also included a range of primary metabolites such as carbohydrates, amino acids, fatty acids and miscellaneous organic acids.

The partial least squares regression analysis showed that 39 from the observed compounds were significantly associated with the outcome of the parasitism and 27 with the classification of the plant material in herbivore resistant or susceptible based on data from Weber et al. 2019 [3]. The successful formation of a mummy was supported by the presence of 16 components (Parasitoid). The case that the beetle averted parasitism and a beetle pupa was formed was positively correlated with the presence of thirteen compounds (Beetle). The pre-pupal mortality of the host larva was linked to eleven compounds (Dead host larva). Plant resistance was positively correlated with 18 and plant susceptibility with 9 compounds.

**Metabolite Group**	**Plant compound**	**Associated with**				
		Mummy	Beetle pupae	Dead larva	Plant resistance	Plant susceptibility
**Acids (citric acid cycle)**	Aconitic acid		+*	+		
	Citric acid	+*				
	Malic acid					+
	2-oxoglutaric acid				+	
	Succinic acid	+				
**Lipids**	Linolenelaidic acid					
	Nonanoic acid			+		
	Palmitic acid	+*			+	
	Stearic acid		+*		+	

**Amino acids**	4-aminobutyric acid					
	Alanine		+*	+		+
	Arginine	+		+		+
	Asparagine					+
	β-alanine				+	
	β-cyano-L-alanine	+*				
	Glutamic acid					
	Proline		+*		+	
	Pyro-glutamic acid					
	Serine					
	Threonine			+		
**Phenolics**	4-hydroxybenzoic acid	+*			+	
	3,4-dihydroxybenzoic acid	+*		+	+	
	Caffeic acid		+*	+		
	Catechin				+	
	Ellagic acid					+
	Shikimic acid				+	
**Terpenoids**	β-sitosterol					+
	Dehydroabietic acid			+		
**Carbohydrates**	Ascorbic acid					+
	β-glucose					+
	Dehydroascorbic acid dimer	+*				
	Erythritol	+				
	Fructose			+		
	Fucose / rhamnose					
	Galacturonic acid	+*				
	Glyceric acid	+*				
	Glucose-6-P					
	Inositol (myo)			+		
	Inositol (scyllo)				+	
	Lyxose / xylose					
	Maltose		+*			
	Melibiose					
	Raffinose					
	Sucrose	+*				
	Tartaric acid		+*		+	
	Trehalose					
	Xylose /arabinose					
	Xylitol /arabitol		+*			
**Structure classified**	NA_carboxylic acid					
**MS tags**	NA_erythrono-1,4-lactone	+*				

	NA_tetraose					
	NA_homoserine					+
	NA_erythrose					
	NA_Pentose					
	NA_Pentose					
	NA_L-threonic acid_1537					
	NA_L-threonic acid_1554					
	NA_sugar					
	NA_lyxonic acid					
	NA_inositol					
	NA_Phytol isomers		+*			
	NA_glyceryl-glycoside TMS ether	+*				
	NA_epicatechin [-O]				+	
**Non-identified MS tags**	NA_ri1392_mz276				+	
	NA_ri1410_mz228		+*			
	NA_ri1414_mz275			+		
	NA_ri1430_mz321				+	
	NA_ri1526_mz393	+*			+	
	NA_ri1610_mz257					
	NA_ri1622_mz129					
	NA_ri1655_mz301		+*			
	NA_ri1658_mz244					
	NA_ri1678_mz244					
	NA_ri1746_mz231		+*			
	NA_ri2017_mz449				+	
	NA_ri2283_mz330					
	NA_ri2445_mz183	+*			+	
	NA_ri2539_mz475		+*			

* Compound retains significant association (i.e., the coefficient CI does not include zero) following adjustment made for multiple tests. See Methods for details.

References

1. A, J. *et al.* Extraction and GC/MS analysis of the human blood plasma metabolome. *Anal. Chem.* **77**, 8086–8094 (2005).
2. Schauer, N. *et al.* GC-MS libraries for the rapid identification of metabolites in complex biological samples. *FEBS Lett.* **579**, 1332–1337 (2005).
3. Weber, D., Egan, P. A., Muola, A. & Stenberg, J. A. Genetic variation in herbivore resistance within a strawberry crop wild relative (Fragaria vesca L.). *Athropod - Plant - Interact.* **14**, 31–40 (2020).
